# Gastric antral defect closure after endoscopic submucosal dissection with the reopenable clip-over-the-line method using a multibending scope

**DOI:** 10.1055/a-2107-2474

**Published:** 2023-07-11

**Authors:** Tatsuma Nomura, Shinya Sugimoto, Taishi Temma, Jun Oyamada, Keiichi Ito, Akira Kamei, Noriya Uedo

**Affiliations:** 1 Department of Gastroenterology, Ise Red Cross Hospital, Ise, Mie, Japan; 2 Department of Gastroenterology, Mie Prefectural Shima Hospital, Shima, Mie, Japan; 3 Department of Gastrointestinal Oncology, Osaka International Cancer Institute, Osaka, Japan

Mucosal defect closure after gastric endoscopic submucosal dissection (ESD) is challenging because of the thick mucosal and muscle layers 1 ; it is particularly difficult in the gastric antrum. When a conventional endoscope is pushed distally to access the antrum, the greater curvature is stretched. This widens the mucosal defect and stretches the muscle layer, making closure of the defect difficult ( Fig. 1 ). Herein, we present a multibending scope technique that allows antral defect closure using the reopenable clip-over-the-line method (ROLM) to avoid stretching the muscle layer 2 .

**Fig. 1:**
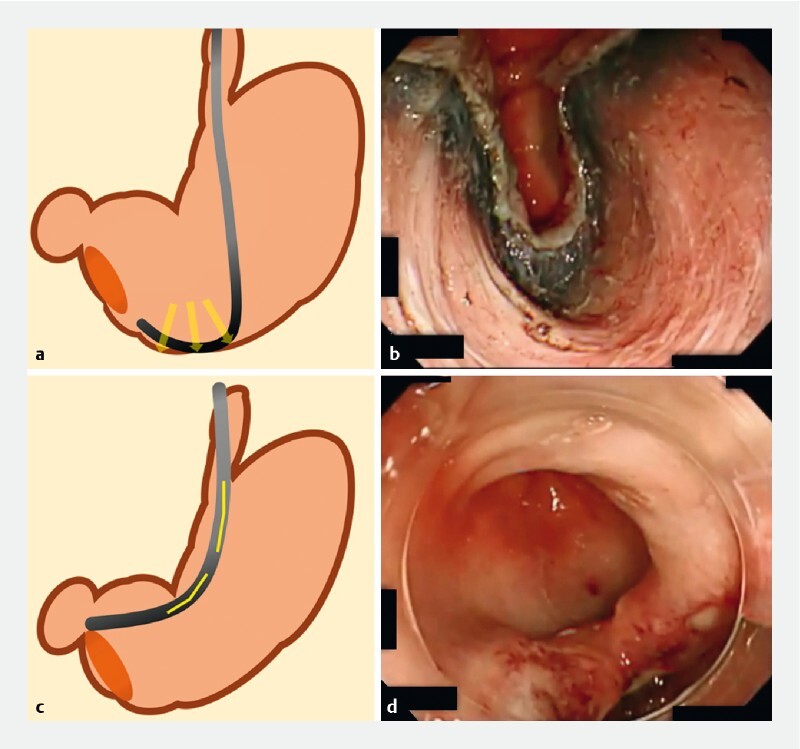
Comparison of mucosal defect closure in the gastric antrum using: **a, b** a conventional upper gastrointestinal scope, which stretches the muscle layer of the defect owing to the stretching of the greater curvature; **c, d** a multibending scope, which touches the greater curvature only slightly and does not stretch the muscle layer of the defect.

A 60-mm mucosal defect was left after gastric ESD in our patient ( Fig. 2 ; Video 1 ). A multibending scope (GIF-2TQ260M; Olympus) was used with the second (top) wheel adjusted to a slightly upward position. Consequently, it was possible to confirm the anal side of the defect edge endoscopically, without stretching the muscle layer. Defect closure was performed using the ROLM, a defect closure technique using a line and reopenable clip (SureClip 8 mm, 16 mm; MicroTech, Nanjing, China) 3 . First, a clip with a line secured to the tooth was placed at the anal edge of the defect through the working channel. The line was passed through a hole in the tooth of the next reopenable clip, before it was also introduced into the working channel. This clip was placed on the contralateral defect edge and nearby muscle layer, with the line tightened over the closed clip before it was deployed. The same steps were repeated to gradually close the defect. Two ROLMs were required for complete defect closure in this case, and the remaining line was cut with a new loop cutter (FS-5L-1; Olympus) 4 . The patient was discharged, without experiencing any adverse events.

**Fig. 2:**
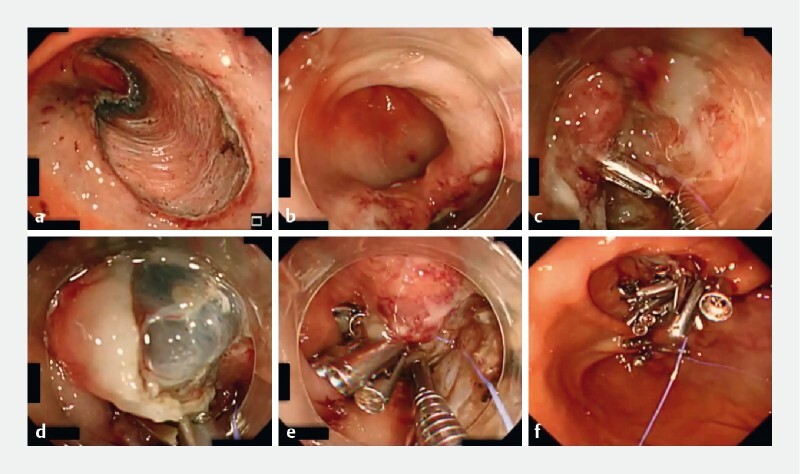
Endoscopic images of defect closure after endoscopic submucosal dissection (ESD) in the gastric antrum using a multibending scope showing: **a** appearance of the 60-mm mucosal defect after ESD with a conventional upper gastrointestinal scope; **b** the same defect viewed with a multibending scope with degassing; **c** placement of the first clip with a line; **d** placement of a clip with a line previously threaded through the tooth on the contralateral mucosal defect edge and muscle layer; **e** defect closure, with the muscle layer folded over, after the placement of further clips threaded onto the line on the contralateral defect edge and muscle layer; **f** complete closure of the mucosal defect.

**Video 1** Defect closure after endoscopic submucosal dissection in the gastric antrum with the reopenable clip-over-the-line method using a multibending scope.


In conclusion, a multibending scope is effective for closing antral mucosal defects because it prevents stretching of the gastric muscle layer.

Endoscopy_UCTN_Code_TTT_1AO_2AG

## Notes

Endoscopy E-Videos

https://eref.thieme.de/e-videos
*E-Videos* is an open access online section of the journal *Endoscopy* , reporting on interesting cases and new techniques in gastroenterological endoscopy. All papers include a high-quality video and are published with a Creative Commons CC-BY license. Endoscopy E-Videos qualify for HINARI discounts and waivers and eligibility is automatically checked during the submission process. We grant 100% waivers to articles whose corresponding authors are based in Group A countries and 50% waivers to those who are based in Group B countries as classified by Research4Life (see: https://www.research4life.org/access/eligibility/ ). This section has its own submission website at https://mc.manuscriptcentral.com/e-videos
